# Hexane Extracts of *Calophyllum brasiliense* Inhibit the Development of Gastric Preneoplasia in *Helicobacter felis* Infected INS-Gas Mice

**DOI:** 10.3389/fphar.2017.00092

**Published:** 2017-02-27

**Authors:** Larissa M. S. Lemos, Fabio Miyajima, Geovane R. C. Castilho, Domingos Tabajara O. Martins, D. Mark Pritchard, Michael D. Burkitt

**Affiliations:** ^1^Gastroenterology Research Unit, Department of Cellular and Molecular Physiology, Institute of Translational Medicine, University of LiverpoolLiverpool, UK; ^2^Department of Basic Sciences in Health, Federal University of Mato GrossoMato Grosso, Brazil; ^3^Department of Molecular and Clinical Pharmacology, Institute of Translational Medicine, University of LiverpoolLiverpool, UK; ^4^Group of Neuropharmacology, Drug Research and Development Center, Federal University of CearáFortaleza, Brazil

**Keywords:** *Helicobacter*, preneoplasia, chromanones, chemoprevention, stomach neoplasms

## Abstract

**Objectives:** Indigenous Latin American populations have used extracts from *Calophyllum brasiliense*, a native hardwood, to treat gastrointestinal symptoms for generations. The hexane extract of *Calophyllum brasiliense* stem bark (HECb) protects against ethanol-mediated gastric ulceration in Swiss–Webster mice. We investigated whether HECb inhibits the development of gastric epithelial pathology following *Helicobacter felis* infection of INS-Gas mice.

**Materials and Methods:** Groups of five male, 6-week-old INS-Gas mice were colonized with *H. felis* by gavage. From 2 weeks after colonization their drinking water was supplemented with 2% Tween20 (vehicle), low dose HECb (33 mg/L, lHECb) or high dose HECb (133 mg/L, hHECb). Equivalent uninfected groups were studied. Animals were culled 6 weeks after *H. felis* colonization. Preneoplastic pathology was quantified using established histological criteria. Gastric epithelial cell turnover was quantified by immunohistochemistry for Ki67 and active-caspase 3. Cytokines were quantified using an electrochemiluminescence assay.

**Results:** Vehicle-treated *H. felis* infected mice exhibited higher gastric atrophy scores than similarly treated uninfected mice (mean atrophy score 5.6 ± 0.87 SEM vs. 2.2 ± 0.58*, p* < 0.01). The same pattern was observed following lHECb. Following hHECb treatment, *H. felis* status did not significantly alter atrophy scores. Gastric epithelial apoptosis was not altered by *H. felis* or HECb administration. Amongst vehicle-treated mice, gastric epithelial cell proliferation was increased 2.8-fold in infected compared to uninfected animals (*p* < 0.01). Administration of either lHECb or hHECb reduced proliferation in infected mice to levels similar to uninfected mice. A T_h_17 polarized response to *H. felis* infection was observed in all infected groups. hHECb attenuated IFN-γ, IL-6, and TNF production following *H. felis* infection [70% (*p* < 0.01), 67% (*p* < 0.01), and 41% (*p* < 0.05) reduction vs. vehicle, respectively].

**Conclusion:** HECb modulates gastric epithelial pathology following *H. felis* infection of INS-Gas mice. Further studies are indicated to confirm the mechanisms underlying these observations.

## Introduction

Gastric cancer is the third commonest cause of cancer death worldwide (Ferlay et al., 2013). Over 80% of patients with primary gastric adenocarcinoma have evidence of prior exposure to *Helicobacter pylori*. Curative treatment for gastric cancer relies on surgical or endoscopic resection of lesions, however, many patients present late in the disease process and hence cannot be offered curative therapy.

Established chemotherapeutic agents are available for patients with gastric cancer, but their efficacy is limited (Bauer et al., 2015). Another strategy that could be employed to reduce the burden of gastric cancer would be to develop chemopreventative strategies that retard the development of gastric cancer in at risk populations. Currently the only effective treatment strategy to achieve this is to eradicate *H. pylori*, but this strategy is becoming more challenging due to the emergence of antibiotic resistant organisms (Shiota et al., 2015), and is relatively ineffective in people who have established preneoplastic pathology (Ford et al., 2014), therefore novel therapeutic agents are needed. As 70% of novel chemotherapeutic agents are derived from plant materials (Newman and Cragg, 2012), the extraction and characterization of novel, naturally occurring compounds is an important strategy for the identification of potentially important new drugs.

*Calophyllum brasiliense* Cambessédes is a tropical hardwood tree of the Calophyllaceae family native to Latin America’s rainforests (Mesia-Vela et al., 2001; The Angiosperm Phylogeny Group, 2016). Many parts of this tree, including the latex that exudes from its bark, have been used in folk medicine to treat a variety of symptoms, including those associated with the gastrointestinal tract (Corrêa, 1978; Reyes-Chilpa et al., 2006; Neto, 2012). The hexane extract of *C. brasiliense* stem bark (HECb) has been shown to protect against models of acute gastric ulceration in Swiss–Webster mice and Wistar rats. The majority of this extract is composed of two chromanones, Brasiliensic acid and Isobrasiliensic acid, these agents have been shown to contribute at least part of the gastroprotective activity of HECb (Lemos et al., 2012).

As HECb and its chromanone fractions influence the development of gastric ulceration, we hypothesized that these agents may also influence the outcome of chronic *Helicobacter* infection, and may modulate the development of gastric cancer. To determine whether this is the case we adopted the established INS-Gas mouse/*H. felis* induced gastric pre-neoplasia model. In this model, constitutively hypergastrinaemic INS-Gas mice are colonized with *H. felis* for 6 weeks. Animals develop marked gastritis with atrophy and early pre-neoplastic lesions identifiable in the gastric corpus of infected mice (Wang et al., 2000; Thomson et al., 2012; Burkitt et al., 2017). We have used this model to characterize how HECb administration influences gastric pre-malignancy and gastric cell turnover.

## Materials and Methods

### Botanical Material

The stem bark of *C. brasiliense* Cambess. was collected in June 2010 by LMSL (authorization number 22698-1 Ministério do Meio Ambiente, Brazil), at the source of the Coxipó River (S15°38′40.8′′, W056°03′05.6′′), Cuiabá, MT, Brazil. A voucher specimen (# 37993) was deposited at the Herbarium of Federal University of Mato Grosso (UFMT), Brazil, and was identified by Harri Lorenzi M.Sc., Instituto Plantarum de Estudos da Flora, Nova Odessa, SP, Brazil. The preparation of HECb, as well as brasiliensic (Bras. acid) and isobrasiliensic acid (Isobras. acid) isolation process, were as previous described (Lemos et al., 2016).

### Animals

All animal procedures were performed at the University of Liverpool with UK Home Office approval. *In vivo* experiments were performed in male INS-Gas mice on the FVB/N (Wang et al., 1993) background bred and maintained at the University of Liverpool Biomedical Services Unit. Primary gastric gland cultures were generated from male C57BL/6 mice acquired from Charles River, Margate, UK.

### *Helicobacter felis* Colonization Experiments

*Helicobacter felis* (ATCC 49179) was cultured for 72–96 h at 37°C on Columbia chocolate agar plates in a microaerophilic environment generated by Campygen atmosphere generating packs in an anaerobic jar (all Oxoid, Basingstoke, UK). For colonization of mice, the organism was harvested into tryptone soy broth and bacterial density was estimated by optical density at 600nm. An estimated bacterial density in excess of >10^8^ CFU/mL was required to progress to gavage.

Groups of at least five male INS-Gas aged 6 weeks were administered 0.5 ml *H. felis* suspension by oro-gastric gavage on three occasions over one week. Successful *H. felis* colonization was confirmed 2 weeks after the final gavage procedure by quantitative PCR for *FlaA* in fecal DNA (Duckworth et al., 2015b). At this time, drinking water was supplemented with 2% Tween 20, HECb 33 mg/L (lHECb, approximately 10 mg/kg/day) or HECb 133 mg/L (hHECb, approximately 40 mg/kg/day) and made available *ad-libitum*. Equivalent uninfected control groups were also studied. Animals were culled by cervical dislocation 6 weeks after *H. felis* colonization. Corpus and antrum mucosal samples were taken for histopathology and immunohistochemistry studies. The remainder of gastric tissue was homogenized in PBS with protease inhibitor for quantification of cytokines by electrochemiluminescent assay.

### Histological Procedures

Gastric tissues were fixed in 4% formalin in PBS for a minimum of 12 h, processed into paraffin wax embedded blocks by standard methods and sectioned at 4 μm thickness for all staining techniques. Corpus and antrum were stained with hematoxylin and eosin (HE) for histopathological evaluations. Immunohistochemical analysis of corpus was also performed.

### Immunohistochemistry

Gastric corpus mucosa was labeled by immunohistochemistry for proliferation (Ki67 primary antibody, AbCam, Cambridge, UK), apoptosis (cleaved caspase 3, AF835, R and D Systems, Minneapolis, MN, USA), and tyrosine 204 phosphorylation state specific ERK (sc-7383, SantaCruz Biotechnology, Dallas, TX, USA) by immunohistochemistry. All primary antibodies were raised in rabbit and were visualized using the Impress HRP system (Vector laboratories, Peterborough, UK).

### Quantitative Histological Methods

Histological tissue sections were scored by an investigator blinded to sample identity, using a modified visual analog scale (Rogers, 2012). To quantify cell numbers in the gastric corpus mucosa, 10 areas per mouse, with well oriented gastric glands, forming a well visualized epithelial monolayer were chosen. Ki67 scoring was performed using a 10 mm × 10 mm eyepiece graticule divided into 1 mm squares which was overlapped along the chosen area using a ×40 objective. Number of positive cells per square were recorded as previously described (Burkitt et al., 2013). Apoptotic and ERK phosphorylation events were scored by examining the number of positively stained cells in 10 high powered fields per section, using a ×63 objective. All results were expressed as mean ± SEM.

### Electrochemiluminescence Immunoassay Analysis

Twelve cytokines were measured in the same samples of gastric homogenate of mice infected or not with *H. felis* by multiplexed electrochemiluminescence cytokine immunoassays (Meso Scale Discovery, Gaithersburg, MD, USA). Specifically, these were a Th1/Th2 standard 10-plex panel consisting of IFN-γ, IL-1β, IL-2, IL-4, IL-5, KC-GRO, IL-10, IL-12 p70, IL-13, and TNF. In addition, simplex IL-17 and IL-23 assays were performed in parallel, as Th17 responses are strongly associated with *Helicobacter* infections. The part of the stomach between corpus and antrum of *H felis* infected and uninfected INS-Gas mice was homogenized twice in PBS with protease inhibitors (SigmaFast, Sigma–Aldrich, UK), using the TissueLyserII (QIAGEN, Tokyo, Japan) at 25 Hz for 3 min. After centrifugation (4°C, 12,000 rpm, 10 min), supernatants were transferred to a clean tube, and stored at −80°C until use. Immediately before analysis, samples were clarified by further centrifugation (4°C, 12,000 rpm, 10 min). Electrochemiluminescence analysis was performed according to the manufacturer’s instructions. A standard curve for each analyte was curve-fitted and allowed determination of the concentration in pg cytokine/mL.

### Murine Primary Gastric Gland Cultures

Gastric epithelial cultures were generated as previously described (Duckworth et al., 2015a) and were maintained in 12-well tissue culture plates on glass cover slips (Appleton Woods, Selly Oak, UK) that contained 1.0 mL/well DMEM-Ham’s F-12 mix (Sigma–Aldrich), 10% fetal calf serum (Invitrogen, Paisley, UK), 1.25% L-glutamine (Sigma–Aldrich), and 1% antibiotic/antimycotic mixture (Sigma–Aldrich). Following digestion and plating, glands were maintained at 37°C in a humidified environment containing 5% CO_2_ for 24 h. Media was changed to fresh complete media after a further 24 h. Forty-eight hours after initial plating, cells were treated with HECb, brasiliensic and isobrasiliensic acids (12.5 to 100 μg/mL) for 24 h, 2 h before fixation EdU was added to the culture media. Cells were fixed in 2% formaldehyde for 30 min followed by three washes in PBS. Treatments were repeated a minimum of four times using glands extracted from a different mouse on each occasion.

### Immunofluorescence

Primary glands were immunolabeled for cleaved caspase 3 and EdU. Two hours before the end of treatment, 200 μL of treatment media was replaced by 200 μL of 10 μM EdU and incubated to complete the treatment. Cultures were washed and fixed with 2% paraformadehyde in PBS for 30 min. Following fixation, EdU intercalation was labeled using the Click-iT EdU Alexa Fluor 594 Imaging Kit (Invitrogen, Paisley, UK) as per protocol. Subsequently non-specific protein binding was blocked with 10% goat serum and apoptotic cells were labeled with a rabbit anti-cleaved caspase 3 antibody (AF835, Cell Signalling, Beverly, MA, USA) and visualized with Alexa fluor 488 conjugated donkey anti-rabbit immunoglobulins (Invitrogen). Coverslips were mounted with Vectashield with DAPI (Vector Labs). Slides were observed using a standard Nikon fluorescent microscope, and proliferative and apoptotic events were quantified as previously described (Duckworth et al., 2015a).

### Human Gastric Cancer Cell Culture

The human gastric adenocarcinoma cell line AGS (ATCC CRL 1739) were grown in complete medium, consisting of Dulbecco’s Modified Eagle’s medium (DMEM) supplemented with 10% fetal calf serum, 1% L-glutamine and 1% penicillin/streptomycin at 37°C in 5% CO_2_ atmosphere with humidity.

### Flow Cytometry

AGS cells (10^6^ cells/well) were seeded in 12-well plates, then treated or not with HECb, brasiliensic, and isobrasiliensic acids (12.5, 25, and 50 μg/mL) for 24 h. Cells were harvested, washed with phosphate-buffered saline (PBS), fixed with cold 70% ethanol and kept at –20°C until use. Cells were washed three times with PBS and stained with a solution of ribonuclease A (R4875, Sigma–Aldrich, Sao Paulo, BR, USA) at 50 μg/mL and propidium iodide (P4170, Sigma–Aldrich, Sao Paulo, BR, USA) at 20 μg/mL in PBS for 90 min, cell cycle distribution was determined by flow cytometric analysis using a BD Accuri™ C6 (BD, Piscataway, NJ, USA).

### Western Blotting and Blot Densitometry

AGS cells (2 × 10^6^ cells/well) were seeded in 6-well plates, pretreated with HECb (12.5, 25, and 50 μg/mL) and a highly selective MEK1 inhibitor (PD98059) at 10 mM for 24 h. Cultures were subsequently infected with *H. pylori* at a MOI of 300:1 for 1 h. After incubation, cells were lyzed in ice-cold RIPA buffer supplemented with protease cocktail and phosphatase inhibitors (Sigma Fast, 10 mM sodium orthovanadate, 10 mM sodium pyrophosphate, and sodium fluoride 100 mM).

Protein lysates were subjected to SDS-PAGE before being immobilized onto nitrocellulose membranes (Biorad, USA). After transfer, membranes were blocked (20 mM Tris-HCl, pH 7.4, 125 mM NaCl, 0.2% Tween 20, 1% bovine serum albumin, 3% non-fat milk) for 1 h at room temperature and incubated for 4 h at 4°C with specific primary antibodies: p-ERK1/2 and β-actin (as above, Santa Cruz Biotechnology, Dallas, TX, USA). Blots were incubated with secondary antibody rabbit anti-mouse IGG-HRP (sc-358914, Santa Cruz Biotechnology, Dallas, TX, USA) and immunoreactive bands were visualized by chemiluminescence (ECL Amersham, USA) and detected with ChemiDoc XRS system™ software and subsequently analyzed with Image Lab™ (Biorad, Irvine, CA, USA).

### Statistical Analysis

Statistical analysis was performed using GraphPad Prism 6 software. Data represent mean ± SEM. Comparisons were made using 1-way or 2-way ANOVAs and Tukey’s or Sidak’s *post hoc* analysis as appropriate. *p* < 0.05 was considered significant.

## Results

### HECb Protects against *H. felis* Induced Pre-neoplasia in INS-Gas Mice

To determine whether the administration of HECb affected the outcome of *H. felis* induced gastric pre-neoplasia, groups of five 6-week old male INS-Gas mice were infected with *H. felis* by oro-gastric gavage, two weeks later drinking water was supplemented with hHECb, lHECb, or vehicle (2% Tween 20). This treatment was maintained until the end of the procedure 6 weeks after the final dose of *H. felis*. At the end of the procedure, animals were culled and gastric epithelial tissues prepared for quantitative histopathology and electrochemiluminescent cytokine analysis. Control groups that underwent changes to their water supply, but did not receive *H. felis* were also maintained.

**FIGURE 1:**
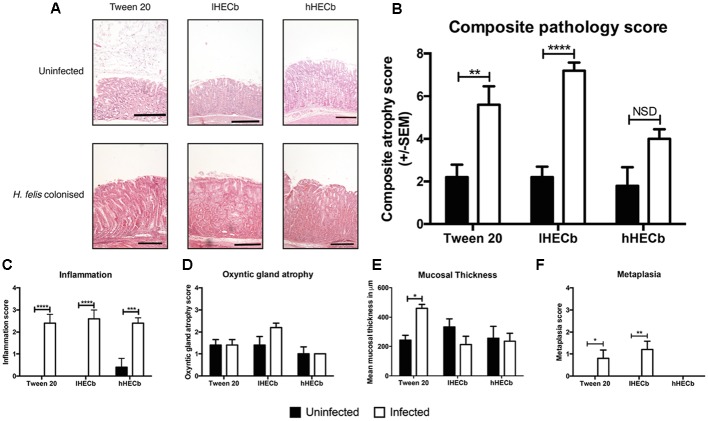
**Evaluation of gastric corpus pathology of INS-Gas mice infected or not with *Helicobacter felis* for 6 weeks and treated with 33 mg/L (lHECb) or 133 mg/L (hHECb) *ad libitum* for the final 4 weeks. (A)** Representative photomicrographs of HE-stained sections of gastric corpus, scale bar = 50 μm. Histopathologic scoring results of **(B)** composite atrophy pathology **(C)** inflammation **(D)** oxyntic gland atrophy **(E)** mucosal thickness, and **(F)** metaplasia. Two-way ANOVA followed by Sidak’s multiple comparison *post hoc* test. All data are mean ± SEM of five mice. ^∗^*p* < 0.05, ^∗∗^*p* < 0.01, ^∗∗∗^*p* < 0.001, ^∗∗∗∗^*p* < 0.0001 vs. uninfected mice with the same treatment.

Pathological gastric lesions were quantified using an established visual analog scoring tool. Uninfected animals had low combined pathology scores (mean score 2.2 ± 0.58), with no significant differences observed in mice exposed to HECb compared to those receiving vehicle. The administration of *H. felis* led to the development of marked gastric corpus pathology in the vehicle group (5.6 ± 0.87). lHECb appeared to have no impact on development of gastric corpus neoplasia at this timepoint, with similar composite pathology scores (7.2 ± 0.37) compared to vehicle treated, *H.* felis infected mice. In contrast, composite pathology scores were partially attenuated in animals co-administered hHECb (4.0 ± 0.45) (**Figures 1A,B**). At this time-point, treatment with HECb had no discernable effect on inflammatory cell infiltration or parietal cell loss (**Figures 1C,D**), however, the gastric mucosa was 1.9 times thicker in vehicle treated *H. felis* infected mice compared to vehicle treated uninfected mice (*p* < 0.05). In HECb treated mice *H. felis* infection did not significantly alter mucosal thickness (**Figure 1E**), and mucous metaplasia was decreased on morphological scoring criteria in *H. felis* infected mice receiving hHECB (**Figure 1F**).

**FIGURE 2:**
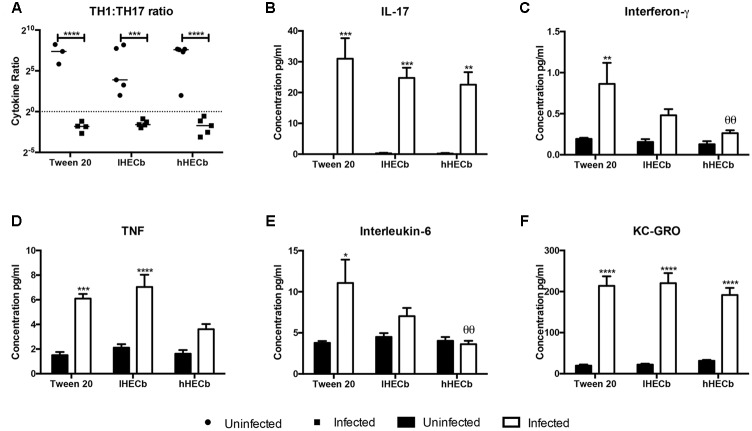
**Effect of hexane extract of *Calophyllum brasiliense* stem bark (HECb) on cytokine abundance in homogenate from the stomachs of INS-Gas mice infected or not with *H. felis* for 6 weeks and treated with 33 mg/L (lHECb) or 133 mg/L (hHECb) *ad libitum* for the final 4 weeks, by electrochemoluminescence assay. (A)** Th1 and Th17 ratio response, abundance of **(B)** IL-17, **(C)** IFN-γ, **(D)** TNF, **(E)** IL-6, and **(F)** KC-GRO in gastric tissue. Two-way ANOVA followed by Tukey’s multiple comparison *post hoc* test. All data are means ± SD of 5 mice. ^∗^*p* < 0.05, ^∗∗∗^*p* < 0.001, ^∗∗∗∗^*p* < 0.0001 vs. uninfected mice with the same treatment, 𝜃𝜃*p* < 0.01 vs. Tween 20 control group with the same infection status.

To further characterize the gastric epithelial immune response to *H. felis* infection in this model mucosal cytokine abundance was determined by electrochemiluminescent assay. Colonization with *H. felis* induced a Th17 polarized immune response, as previously demonstrated in this and other mouse models of *Helicobacter* induced gastric pre-neoplasia (**Figures 2A,B**).

Administration of either vehicle or HECb did not alter this overall response, however, there were subtle changes in the abundance of individual cytokines. Amongst *H. felis* infected mice IFN-γ, TNF, and IL-6 were all less abundant (3.3-fold, 3.1-fold, and 1.7-fold, respectively) in animals administered hHECb compared to those treated with vehicle. Treatment with lHECb also induced an apparent, though statistically not-significant, reduction in IFN-γ and IL-6 abundance, supportive of a dose response effect for HECb on production of these cytokines. lHECb had minimal impact on TNF abundance compared to vehicle (**Figures 2C–E**). The abundance of KC-GRO, a mouse homolog for IL-8, in vehicle treated mice was 10.9-fold more abundant in mice colonized with *H. felis*, compared to the uninfected group, this cytokine was unaffected by the administration of HECb (**Figure 2F**). These observations suggest that treatment with HECb minimally attenuates the inflammatory response induced by *H. felis*.

### HECb Influences Gastric Epithelial Remodeling by Altering Epithelial Cell Turnover in Response to *H. felis In vivo*

**FIGURE 3:**
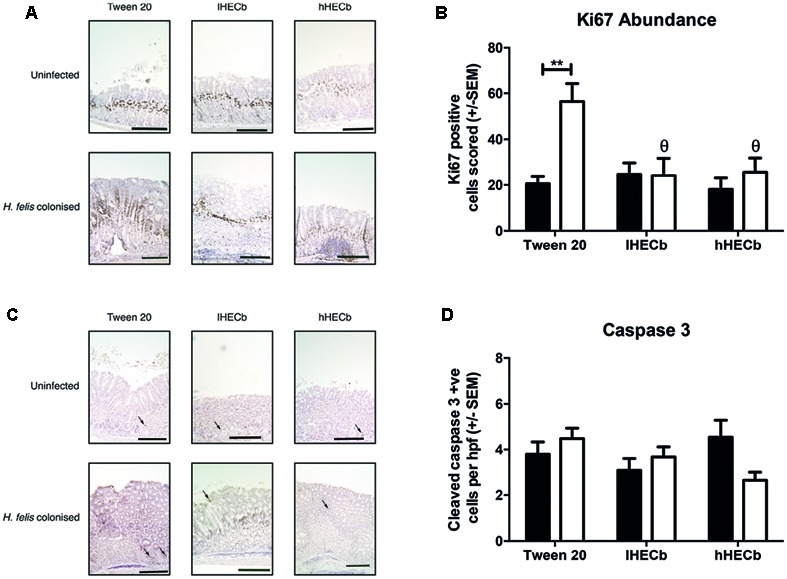
**Effect of HECb on cell turnover in gastric corpus of INS-Gas mice infected or not with *H. felis* for 6 weeks and treated with 33 mg/L (lHECb) or 133 mg/L (hHECb) *ad libitum* for the final 4 weeks. (A)** Representative photomicrographs of proliferating cells immunostained with Ki67 and **(B)** graph showing Ki67 positive cells scored, scale bars 50 μm **(C)** representative photomicrographs of apoptotic cells immunostained for cleaved caspase-3 and **(D)** graph showing the number of cleaved caspase 3 positive cells per high powered fields. Two-way ANOVA followed by Tukey’s multiple comparison *post hoc* test. All data are mean ± SEM of five mice. ^∗∗^*p* < 0.01 vs. uninfected mice with the same treatment, 𝜃*p* < 0.05 vs. Tween20 control group with the same infection status.

The observation that *H. felis* induced metaplasia was less abundant in mice treated with hHECb led us to hypothesize that HECb treatment might influence epithelial remodeling, either impacting de-differentiation of mature cell lineages, or influencing epithelial cell turnover. To characterize this, quantitative histology was used to determine the number of Ki67 positive proliferating cells (**Figure 3A**), and cleaved caspase 3 positive (**Figure 3C**) apoptotic cells in the gastric corpus mucosa of mice.

A mean apoptotic index of 3.8 (±0.53) cells per high powered field (hpf) was demonstrated in vehicle treated, uninfected mice. This did not change significantly following *H. felis* infection, or administration of HECb (**Figure 3D**). In contrast *H. felis* infection had a profound impact on abundance of Ki67 immunopositive cells. Uninfected mice treated with Tween20 had a proliferation index of 20.5 (±3.2), which was similar to the proliferation index of uninfected mice treated with HECb. Following *H. felis* infection vehicle treated mice exhibited a 2.8-fold increase in proliferative index (*p* < 0.01, **Figure 3B**). When HECb was administered at either dose Ki67 abundance was significantly lower in infected mice compared to vehicle treated controls (2.3-fold and 2.2-fold reductions for hHECb and lHECb, respectively, *p* < 0.01), leading to proliferation indices similar to those seen in uninfected mice.

### HECb Induced Suppression of Proliferation is an Epithelial Cell Event

To determine whether the anti-proliferative effect of HECb observed in *H. felis* infected INS-Gas mice was driven purely by its apparently modest influence on inflammation, or through an immune cell independent mechanism, primary cultures of murine gastric glands were generated. In our hands these cultures can be maintained for in excess of 5 days, and have previously been shown to contain cells of each of the major gastric epithelial lineages (Duckworth et al., 2015a).

Cultures were generated from male C57BL/6 mice. On the third day of culture, gastric glands were treated with rising concentrations of HECb or its constituents, brasiliensic acid, and isobrasiliensic acid. Cells were fixed at 24 h. Epithelial cell proliferation was assayed by quantifying the percentage of cells that had intercalated EdU, apoptosis was quantified by immunofluorescence for cleaved caspase-3. Each treatment was performed on cultures derived from four individual mice.

**FIGURE 4:**
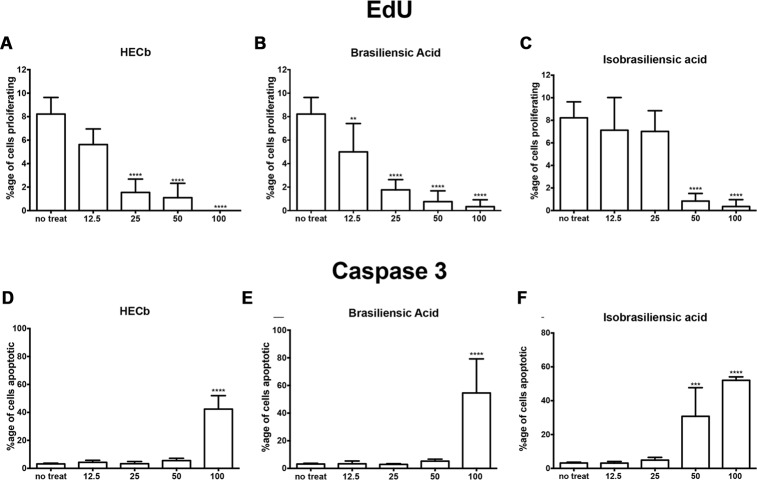
**Effects of HECb on cell turnover of murine primary gastric epithelial cell cultures treated with HECb, brasiliensic acid or isobrasiliensic acid (12.5–100 μg/mL) for 24 h, evaluated by immunofluorescence.** Data expressed as percentage of proliferating cells following **(A)** HECb, **(B)** brasiliensic acid, and **(C)** isobrasiliensic acid and percentage of apoptotic cells following **(D)** HECb, **(E)** brasiliensic acid, and **(F)** isobrasiliensic acid. Two-way ANOVA followed by Tukey’s multiple comparison *post hoc* test. All data are mean ± SD *n* = 4. ^∗∗^*p* < 0.01 ^∗∗∗^*p* < 0.001, ^∗∗∗∗^*p* < 0.0001 vs. untreated cells.

In untreated cultures, 8.2% (±0.58) of cells intercalated EdU into their DNA. At different doses of treatment with HECb, brasiliensic acid, and isobrasiliensic acid all suppressed proliferation (**Figures 4A–C**). Significant suppression of proliferation was observed following treatment with HECb at doses in excess of 25 μg/mL. Brasiliensic acid partially suppressed proliferation at 12.5 μg/mL and had more pronounced effects at doses in excess of 25 μg/mL. Isobrasiliensic acid suppressed proliferation at doses of 50 μg/mL and 100 μg/mL.

All three compounds also induced apoptotic responses. Cytotoxicity in this model occured following treatment with 100 μg/mL of HECb or Brasiliensic acid. 100 μg/mL HECb induced a 13.3-fold increase in apoptosis compared to untreated glands (42% apoptotic cells, *p* < 0.001), whilst 100 μg/mL Brasiliensic acid triggered 55% (*p* < 0.0001) of cells to become apoptotic. Isobarasiliensic acid treatment induced apoptosis at both 50 μg/mL and 100 μg/mL with, respectively, 31 and 52% of cells shown to be apoptotic (**Figures 4D–F**). These observations demonstrate that HECb and its constituents induce cell cycle arrest and apoptosis in untransformed epithelial cell culture, suggesting that there is a direct epithelial effect of these compounds.

To characterize whether the impact of HECb and its constituents on gastric epithelial cell proliferation was an isolated phenomenon in the *ex-vivo* culture setting, or whether the same effects are identifiable in transformed cell lines, the cell cycle dynamics of AGS cells treated with HECb and its constituent chromanones was characterized by propidium iodide FACS analysis. Experiments were repeated a total of 4 times for each treatment.

**FIGURE 5:**
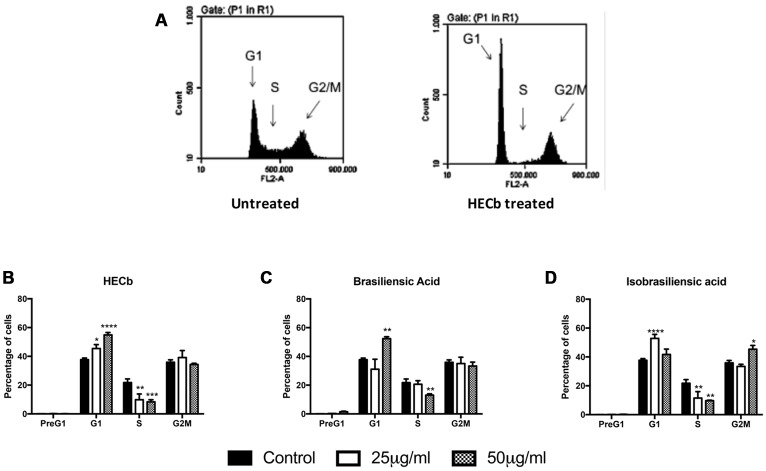
**Effect of HECb and its constituents on cell cycle of transformed gastric epithelial cells.** AGS cells were treated with HECb, brasiliensic acid, or isobrasiliensic acid (25 or 50 μg/mL) or untreated for 24 h and stained with propidium iodide. **(A)** Shows representative plots from untreated and HECb treated cells demonstrating the shift in distribution of cells by cell cycle phase folowing HECb administration. **(B–D)** Show the mean percentage of cells (±SEM) in PreG1, G1, S, and G2M phases of cell cycle, HECb **(B)**, brasiliensic acid **(C)** and isobrasiliensic acid **(A)**. Two-way ANOVA followed by Tukey’s *post hoc* analysis. ^∗^*p* < 0.05; ^∗∗^*p* < 0.01; ^∗∗∗^*p* < 0.001; ^∗∗∗∗^*p* < 0.0001 vs. untreated cells in the same phase of cell cycle.

In untreated AGS cultures, 0.11% (±0.02 %) of cells were identified in the pre-G1 apoptotic phase, 37.7% (±1.14%) were in the G1 phase, 21.8 % (±2.52 %) of cells were in S phase and 35.7% (±1.7 %) were in G2M phase (**Figure 5**). No increase in the proportion of cells in pre-G1 was observed when AGS cells were treated with HECb or its constituents at 25 or 50 μg/mL. This is in keeping with our findings in primary cell culture where apoptosis was not induced when these concentrations were tested.

Hexane extract of *Calophyllum brasiliense* stem bark (HECb) significantly decreased the proportion of cells in S-phase at both 25 and 50 μg/mL (9.9 and 8.3%, of cells in S-phase, respectively, **Figure 5A**), with a reciprocal increase in proportion of cells in G1 (45.5 and 54.9%, *p* < 0.05 and *p* < 0.0001 respectively for 25 and 50 μg/mL HECb). Brasiliensic acid induced a similar reduction in percentage of cells in S-phase at a dose of 50 μg/mL (13.0% of cells in S-phase, *p* < 0.01 **Figure 5B**), with an increase in proportion of cells in G1 observed (52.3%, *p* < 0.01). Both 25 and 50 μg/mL isobrasiliensic acid also reduced the proportion of cells in S-phase compared to control (11.5 and 9.4% of cells in S-phase, respectively, both *p* < 0.01 **Figure 5C**). Intriguingly, however, lower dose isobrasiliensic acid induced an increase in proportion of cells in G1 (52.9 %, *p* < 0.001), similar to that observed in cells treated with either HECb or brasiliensic acid, whilst higher dose isobrasiliensic acid appeared to induce G2M arrest with an increase in the number of cells in this phase (45.4%, *p* < 0.05).

These data demonstrate that HECb and its constituents are capable of inducing cell cycle arrest in transformed cell lines. The evidence that isobrasiliensic acid induced G1 arrest at low dose and G2M arrest at higher doses attests to these compounds potentially acting through more than one mechanism, dependent upon the drug dosing regime.

### HECb Suppresses *Helicobacter* Induced Phosphorylation of ERK *In vitro* and *In vivo*

To further characterize how HECb affects *Helicobacter* induced proliferation, we pre-treated AGS cells with either HECb or the MEK 1 inhibitor PD98025 for 24 h. Subsequently cells were co-cultured with *H. pylori* at a multiplicity of infection of 300:1 for 1 h. A well characterized strain of *H. pylori* was used for these asssays, rather than *H. felis* as the effects of this organism on human cell culture are better characterized than those of *H. felis*, and *H. pylori* infection is more relevant to human disease.

The abundance of p-ERK in whole cell lysates was quantified by Western blotting. All experiments were repeated three times. Blot densitometry was performed to quantify relative expression of p-ERK compared to pan-actin abundance.

**FIGURE 6:**
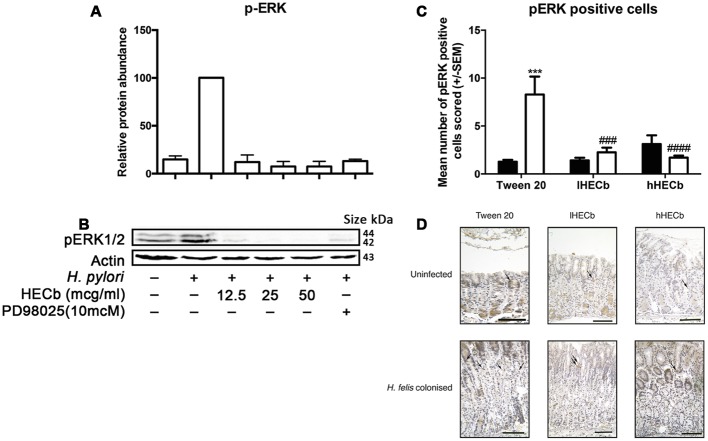
**Effect of HECb on *Helicobacter* induced phosphorylation of ERK *in vitro* and *in vivo.*** AGS cells were pretreated with HECb (12.5–50 μg/mL) for 24 h, and infected with *H. pylori* (MOI 1:300) for 1 h. **(A)** p-ERK1/2 abundance relative to β-actin. One-way ANOVA, followed by Sidak’s *post hoc* test. Data are mean ± SD *n* = 3. ^∗∗∗∗^*p* < 0.0001 vs. untreated, uninfected cells. **(B)** Representative western blotting. Phosphorylation was estimated in relation to the relative amount of the endogenous β-actin control. Each line represents the mean of three independent experiments. **(C)** Effect of HECb on ERK1/2 phosphorylation in gastric corpus of INS-Gas mice infected or not with *H. felis* for 6 weeks and treated with 33 mg/L (lHECb) or 133 mg/L (hHECb) *ad libitum* for the final 4 weeks. Means ± SEM. *N* = 5. ^∗∗∗^*p* < 0.001 vs. uninfected mice with the same treatment, ^##^*p* < 0.01, ^###^*p* < 0.001 vs. Tween 20 control group with the same infection status. **(D)** Representative photomicrographies of ERK1/2 immunostaining, scale bar = 25 μm.

Exposure of AGS cells to *H. pylori* for 1h induced phosphorylation of ERK 1 and 2. When cells were pre-treated with HECb we observed significantly less phosphorylation of ERK at all doses that were administered (**Figures 6A,B**).

To determine whether ERK phosphorylation was also involved in the reduction of gastric epithelial cell proliferation in response to *H. felis* infection, *in vivo* gastric corpus tissue samples from mice infected with *H. felis* or not, and treated with hHECb, lHECb or vehicle were immunostained for p-ERK. The number of cells expressing p-ERK was determined by quantitative immunohistochemistry.

The gastric corpus of vehicle treated, uninfected mice exhibited 1.3 (±0.2) p-ERK positive cells per high power field. Administration of HECb did not significantly influence this in uninfected mice, however, administration of *H. felis* induced a 6.4-fold (*p* < 0.001) increase in p-ERK positive cells in mice treated with vehicle. *H. felis* induced phosphorylation of ERK was almost entirely suppressed by treatment with either of the tested doses of HECb (**Figures 6C,D**).

This suggests that regulation of a classical MAPK pathway may be targeted directly or indirectly by HECb administration both *in vitro* and *in vivo.*

## Discussion

The data presented here provide further evidence that the oral administration of HECb influences the outcome of gastric epithelial injury. These effects were observed in the context of minimal changes in inflammatory phenotype with only a modest reduction in cytokine production in hHECb treated mice and no difference in morphological inflammation. It therefore appears likely that HECb acts predominantly through a protective effect on the gastric epithelium. This is in keeping with previous studies which demonstrated mucosal protection by HECb and some of its fractions during stress or chemically induced gastric ulceration (Sartori et al., 1999). In rats with ethanol induced gastric lesions HECb administration led to the inhibition of malondialdehyde and catalase activity suggesting that this gastroprotective role is, in part, due to an antioxidant effect (Lemos et al., 2012).

The mechanism by which HECb influences gastric epithelial homeostasis remains incompletely understood, however, we have now shown that it suppresses proliferation in gastric epithelial cells both in untreated primary cell culture and in transformed cell lines. In addition, we have shown that *H. felis* induced proliferation is suppressed *in vivo* by this compound. *In vitro* we also demonstrated marked gastric epithelial cell cytotoxicity at higher doses of HECb (100 μg/mL). However, at doses used *in vivo* this was not observed, suggesting that effective pharmacological doses probably did not reach this toxic concentration.

To understand how HECb influences proliferation at a molecular level we have characterized the phosphorylation of ERK. ERK is phosphorylated in response to *Helicobacter* co-culture *in vitro*, whilst administration of HECb suppresses *Helicobacter* associated phosphorylation of ERK. *In vivo* we also observed marked suppression of *Helicobacter* induced phosphorylation of ERK when mice were treated with HECb. This suggests that HECb interacts with the Ras-Raf-MEK-ERK pathway, though it remains unclear whether this is through direct interaction with a pathway member, or whether this effect is secondary to interaction with upstream regulators of the pathway. Further mechanistic studies aiming to characterize the precise interaction of HECb and its constituents with mammalian proteins are indicated to enable us to understand the mechanism of action of this extract.

Due to the complexity of extracting the constituent chromanones from HECb it has not been possible to characterize the effects of either brasiliensic or isobrasiliensic acids on murine pre-neoplastic pathology. However, observations from *ex vivo* and *in vitro* cell culture models suggest that both of these agents are able to influence gastric epithelial cell turnover. In untransformed cells, brasiliensic acid appeared to have the widest potential therapeutic window where epithelial cell proliferation was suppressed, but apoptosis had not been induced (between 12.5 and 100 μg/mL), however, the effect of these doses of brasiliensic acid on the proliferation of transformed cell lines was more modest, and isobrasiliensic acid at doses of 25 and 50 μg/mL were required to induce cell cycle arrest. In this model an increase in apoptosis was not observed at the tested doses. Intriguingly we also demonstrated that high doses of isobrasiliensic acid induced a G2M cell cycle arrest as opposed to the G1 arrest observed following administration of either low dose isobrasiliensic acid, or HECb or brasiliensic acid at any dose tested.

Chromanones synthesized or extracted from diverse sources have previously been assessed and shown to exhibit diverse pharmacological functions [including antimicrobial (Xu et al., 1998; Kanokmedhakul et al., 2002; Cottiglia et al., 2004; do Nascimento et al., 2007; Tanaka et al., 2009), anti-oxidant (Lee et al., 2005) and anti-inflammatory effects (Konieczny et al., 1976), as well as effects on cardiac muscle repolarization (Wang et al., 2014) and coronary artery vasodilation (Nagao et al., 1972)]. This diversity of pharmacological activity supports our cell-cycle data which may suggest divergent mechanisms of action for brasiliensic and isobrasiliensic acids at higher doses.

The differences in drug doses that induce apoptosis and cell cycle arrest suggest that there may be therapeutic windows in which these compounds could be used to induce gastric cell cycle arrest without inducing cytotoxicity. These findings support the need for further studies to investigate whether HECb and its constituents may influence the process of human gastric carcinogenesis.

## Ethics Statement

This study was carried out in accordance with the recommendations of the University of Liverpool Ethical Review Panel. The protocol was approved and licensed by the UK Home Office.

## Author Contributions

LL: Generated primary data, contributed to data analysis, and drafted manuscript. FM: Generated primary data, contributed to data analysis, edited manuscript, and helped secure funding. GC: Generated primary data. DM: Conceived intellectual concept, supervised primary data generation, helped secure funding, and edited manuscript. DP: Conceived intellectual concept, supervised primary data generation, helped secure funding, and edited manuscript. MB: Conceived intellectual concept, generated primary data, supervised primary data generation, led data analysis, drafted manuscript, and helped secure funding.

## Conflict of Interest Statement

The authors declare that the research was conducted in the absence of any commercial or financial relationships that could be construed as a potential conflict of interest.
